# Investigating frequency and causes of injures and mortality from traffic accidents in Tabriz hospitals 

**Published:** 2019-08

**Authors:** Homayoun Sadeghi-Bazargani, Ali Janati, Yalda Mousazadeh

**Affiliations:** ^*a*^Road Traffic Injury Research Center, Tabriz University of Medical Sciences, Tabriz, Iran.; ^*b*^Iranian Center of Excellence in Health Management (IceHM), School of Management and Medical Informatics, Tabriz University of Medical Sciences, Tabriz, Iran.; ^*c*^Iranian Center of Excellence in Health Management (IceHM), School of Management and Medical Informatics, Tabriz University of Medical Sciences, Tabriz, Iran

## Abstract

**Background:**

Traffic accidents are considered as the most common cause of injury and the second cause of death in Iran. The majority of victims are young people. Purposes: Therefore, the purpose of this study was to determine the frequency and causes of injuries and deaths from traffic accidents and factors associated with it in Tabriz hospitals.

**Methods:**

This study was a descriptive study between September 2016 and February 2018. Databases of the emergency medical and forensic medicine of East Azarbaijan Province were used to collect data. Data included the type of vehicle injured and involved persons in accident, the dead’s position at the time of the accident, the location of the death, the part of the body that hit it, the mechanism of damage and the final cause of death. The data was analyzed using the Stata software.

**Results:**

11,238 traffic accident victims were included in this study. The largest number of patients including 7194 patients (72.88%) was transferred to Imam Reza. 71 people were identified as died including 66 (92.96%) male and 5 (7.04%) female. Mean (SD) age of victims was 42.2(2.5). The most of died patients were in Imam Reza hospital with 50 patients. The most of death were related to pedestrians (31 people). The largest number of vehicles involved in crashes was cars with frequency of 36 cases. The most common accident mechanism was the collision with the pedestrians (31 cases). The highest number of death (45) was occurred in the hospital and the lowest death was in the home (1). The greatest number of injuries among died was in head and face with frequency 25 and 35.21%. The most common cause of mortality was head trauma with 31 cases and 43.66 %.

**Conclusions:**

This study provided useful information on the epidemiological information of the traffic accident victims. It helps to designing appropriate interventions in order to prevent traffic accidents and their complications, as well as to minimize injuries in accordance with other relevant organizations.

**Keywords:**

Mortality, Traffic accident, Injury, Hospital, Tabriz

